# Super-enhancers and novel therapeutic targets in colorectal cancer

**DOI:** 10.1038/s41419-022-04673-4

**Published:** 2022-03-11

**Authors:** Qian Liu, Lijuan Guo, Zhiyuan Lou, Xueping Xiang, Jimin Shao

**Affiliations:** 1 Department of Pathology & Pathophysiology, and Cancer Institute of the Second Affiliated Hospital, Zhejiang University School of Medicine, Hangzhou, China; 2 Department of Pathology of Sir Run Run Shaw Hospital, Zhejiang University School of Medicine, Hangzhou, China; 3 Key Laboratory of Disease Proteomics of Zhejiang Province, Key Laboratory of Cancer Prevention and Intervention of China National Ministry of Education, Zhejiang University School of Medicine, Hangzhou, China; 4 Cancer Center, Zhejiang University, Hangzhou, China

**Keywords:** Cancer therapy, Tumour biomarkers

## Abstract

Transcription factors, cofactors, chromatin regulators, and transcription apparatuses interact with transcriptional regulatory elements, including promoters, enhancers, and super-enhancers (SEs), to coordinately regulate the transcription of target genes and thereby control cell behaviors. Among these transcriptional regulatory components and related elements, SEs often play a central role in determining cell identity and tumor initiation and progression. Therefore, oncogenic SEs, which are generated within cancer cells in oncogenes and other genes important in tumor pathogenesis, have emerged as attractive targets for novel cancer therapeutic strategies in recent years. Herein, we review the identification, formation and activation modes, and regulatory mechanisms for downstream genes and pathways of oncogenic SEs. We also review the therapeutic strategies and compounds targeting oncogenic SEs in colorectal cancer and other malignancies.

## Facts

- CRC cells form oncogenic SEs through genetic and epigenetic alterations and 3D chromosomal remodeling.
- CRC-related SEs activate the expression of oncogenes via transcriptional and posttranscriptional mechanisms, facilitate immune escape and propel cancer proliferation and metastasis.
- Oncogenic SEs have emerged as attractive targets for novel cancer therapeutic strategies.

## Open questions

- How can cancer-specific and cancer-type-related SEs achieve dynamic assembly?
- More cutting-edge technologies are expected to visualize the function of oncogenic SEs in situ, real-time, and dynamically.
- Next generation of cancer-specific therapeutic drugs may come soon by targeting oncogenic SEs.

## Introduction

Colorectal cancer (CRC) is a malignancy with high morbidity and mortality worldwide [1, 2]. There are diverse underlying pathogenic mechanisms in CRC, including somatic mutations, genetic instability, gene fusions, and epigenetic alterations [3–5]. Radical resection is the primary option for CRC treatment, while chemoradiotherapy, targeted therapy, and immunotherapy are recognized as adjuvant therapies or treatments for unresectable CRC. The high death rate of CRC patients is mainly attributed to the high rate of metastasis and recurrence and the shortage of novel effective therapies [6]. Further clarification of disease mechanisms and development of novel potent therapeutics are still urgently needed tasks for efficient CRC treatment.

Gene transcription is a complex and highly coordinated process. Transcription factors (TFs), cofactors, chromatin regulators, RNA polymerase II (Pol II), and related transcriptional machinery directly or indirectly bind to transcriptional regulatory elements, including promoters, enhancers and super-enhancers (SEs), thereby regulating the expression of target genes [7–11]. In 2013, Young and colleagues used the term ‘super-enhancers’ to describe large clusters of enhancers that drive the transcriptional expression of genes that define cell identity [12–14]. The researchers created a catalog of SEs and their associated genes in a broad spectrum of human cell and tissue types, showed that disease-associated variations were especially enriched in the SEs of disease-relevant cell types, and importantly, proposed that cancer cells generally acquired SEs at oncogenes and other genes that play important roles in cancer pathogenesis.

SEs and enhancers can be bound by the same factors, including TFs, coactivators, chromatin regulators, and the RNA Pol II complex. However, SEs differ from typical enhancers mainly in their large size, higher density, and content of transcriptional regulators, thereby having a higher ability to activate transcription and more sensitivity to perturbation [15–19]. While some TF binding sites are located in promoters, the overwhelming majority are in enhancers and SEs [13, 20]. In particular, SEs are densely occupied by chromatin regulators such as bromodomain-containing protein 4 (BRD4), which participates in transcription and epigenetic regulation by binding acetylated lysine residues on target proteins, including histones [21], and via coactivators such as Mediator complex subunit 1 (MED1), one component of the Mediator complex, which contributes to targeting and anchoring the complex to cell type-specific TFs and many nuclear receptors [22]. Furthermore, active SEs are usually enriched with high levels of histone modifications H3K4me1 and H3K27ac. Increasing high-resolution studies on DNA three-dimensional structures [18, 23, 24] support that chromosomal DNA looping achieves physical proximity between SEs/constituent enhancers and promoters, mediated by diverse regulatory factors, to drive highly efficient transcription [25, 26].

The dysregulated transcription of oncogenes and tumor suppressor genes, driven by genetic and epigenetic alterations, plays a vital role in both cancer initiation and progression in all tumor types [27–31]. Tumor cells can form oncogenic SEs in key oncogenes and genes that function in the acquisition of cancer hallmark capabilities as a result of mutations, epigenetic alterations, or chromosomal remodeling, leading to abnormal binding of different or highly active transcriptional regulatory components, thus propelling oncogenic transcription and tumor development [27, 30–32]. Therefore, oncogenic SEs have been vigorously explored as potential novel drug targets for cancer treatment in recent years. In this review, using CRC as an example, we summarize the identification approaches, architecture, activation mechanisms, downstream genes, and related pathogenic pathways of oncogenic SEs, as well as the therapeutic strategies and compounds targeting oncogenic SEs in CRC and other malignancies.

## Identification approaches of SEs in CRC

### Identification of SE profiles in CRC

Identification of SEs at the genome-wide level usually includes three steps[13]: step 1, localizing enhancers based on chromatin immunoprecipitation sequencing (ChIP-seq) enrichment of MED1, H3K27ac, p300, or master TFs; step 2, stitching enhancers and ranking the stitched enhancers through their ChIP-seq signals in the genomic region via the rank ordering of super-enhancers (ROSE) algorithm; and step 3, separating SEs from typical enhancers based on a cutoff value using the ROSE algorithm, which takes into account enhancer ranks and ChIP signals [33].

A genome-wide comprehensive investigation of aberrant SEs in CRC was carried out by Andrea’s laboratory in 2017 [31]. The study identified recurrent CRC-specific gained or lost SEs through H3K27ac ChIP-seq in more than forty genetically diverse CRC specimens compared to normal colonic crypt epithelia, delineating a comprehensive SE atlas in CRC. Subsequently, hypergeometric optimization of motif enrichment (HOMER) analyses [34] revealed that AP-1 and cohesin complex members were enriched in recurrently gained variant enhancer loci, which always activate known oncogenes. Finally, the function of these oncogenic SEs was confirmed via genome editing and pharmacologic inhibition in experimental models of CRC in vitro and in vivo. In addition, RNA-seq has often been used to assist in establishing network relations between SEs and abnormally expressed mRNAs to uncover SE-driven coregulatory mechanisms of targeted genes underlying tumor development and therapeutic opportunities.

### Advanced technologies for the screening and identification of SEs

In recent years, increasing high-throughput sequencing technologies have become powerful tools for genome-wide screening and identification of enhancers and SEs. Histone modification markers such as H3K27ac, TFs, and the transcription cofactor p300, which are enriched in enhancers and SEs, can be captured across the genome by ChIP-seq, which is the most commonly used high-resolution and high-coverage method for the identification of enhancers and SEs. More recently, further advanced technologies have been developed for the identification of enhancers and SEs based on diverse mechanisms (Table 1) [17, 35–52]. All these approaches make it possible to achieve more precise, systematic and comprehensive studies of enhancers and SEs in physiological processes and disease development.

**Table 1:** Identification approaches of SEs in cancers.

Method	Description	Advantage	Disadvantage
ChIP-seq [17, 35]	An approach to detect genome-wide DNA segments interacting with transcription factors and histones.	Low signal noise, high resolution, and genome-wide covering	Unstable data accuracy and high antibody requirements
ChIP-exo [36]	An approach to identify genomic location of DNA-binding proteins at near single-nucleotide accuracy	Stable single-nucleotide resolution and low background noise	Only single binding event can be detected
3C-seq [37]	An approach to detect the DNA-DNA interactions between two chosen transcriptional regulatory elements.	Quantifiable and cheap	Low throughput and not unbiased
4C-seq [38, 39]	An approach to detect genome-wide DNA-DNA interactions with a single chosen genomic location of interest.	High-resolution and few sample	Inefficient because primers are different for each ‘viewpoint’
Hi-C [40, 41]	An approach to detect pairwise contacts between virtually any pair of genomic loci, constructing the 3D structure of chromatin interaction.	Resolve all chromatin conformations	Large amount of sequencing data, poorly specific and low signal-to-background ratio
ChIA-PET [42]	An approach to study genome-wide chromatin interactions mediated by a protein of interest.	Long-range associations related to the protein factor of interest	Few reads of interest genes, low efficiency, and false positives
HiChIP [43]	An approach to analyze protein-directed genome architecture	Few cells requirement, high signal-to-background ratio, and high specificity	Generate the chromatin conformation bound by the protein factor of interest
STARR-seq [44, 45]	An approach to identify transcriptional enhancers and to assess their activity quantitatively by cloning DNA fragments downstream of a core promoter.	Providing genome-wide quantitative enhancer activity maps of any cell type without being affected by the location of the sequences	The possibility of repeated identification because of lack of accurate context markers.
GRO-seq [46]	An approach to map nascent transcripts at the genome-wide scale, providing a reliable measure of transcriptional activity	Determine the relative activity of the transcription site without knowing its location.	GRO-seq can only measure the length of 10-50 bp, which reduces the accuracy of TSS detection
5hmC-seal [47, 48]	An approach for genome-wide 5hmC profiling using chemical conjugation and affinity purification followed by next-generation sequencing	Genome-wide profiles of 5hmC in DNA across broad-scale tissue types with high accuracy and resolution	Expensive
DNase-seq [49]	An approach to identify the location of regulatory regions, based on the genome-wide sequencing of regions sensitive to cleavage by DNase I.	Simple, wide range of applications	Difficult to control digestion conditions, large sample size, and sequence-dependent on DNA cleavage.
FAIRE-seq [50]	An approach for isolating and sequencing nucleosome-depleted regions of the genome.	No sequence-dependent on DNA cleavage and no requirement for the initial state of the cell.	Low signal-to-noise ratios and high background signal.
ATAC-seq [51]	An approach for assaying chromatin accessibility genome-wide	Simple, small sample size and high resolution	Expensive and different optimal number of cells

ChIP-seq Chromatin Immunoprecipitation sequencing, ChIP-exo Chromatin immunoprecipitation with lambda exonuclease, 3C-seq Chromosome conformation capture, 4C-seq Circularized chromosome conformation capture, Hi-C High-throughput chromosome conformation capture, ChIA-PET Chromatin Interaction Analysis by Paired-End Tag Sequencing, HiChIP In situ Hi-C library followed by ChIP, STARR-seq Self-transcribing active regulatory region sequencing, GRO-seq Global run-on sequencing, 5hmC-seal Genome-wide profiling of 5-hydroxylmethylcytosine Sequencing, DNase-seq DNase I coupled to high-throughput sequencing, DNase-seq DNase I coupled to high-throughput sequencing, FAIRE-seq Formaldehyde-assisted isolation of regulatory elements coupled with high-throughput sequencing, ATAC-seq Assay for Transposase Accessible Chromatin using sequencing.

## The formation and activation of SEs in CRC

### Genomic mutations and variations

**Fig. 1:**
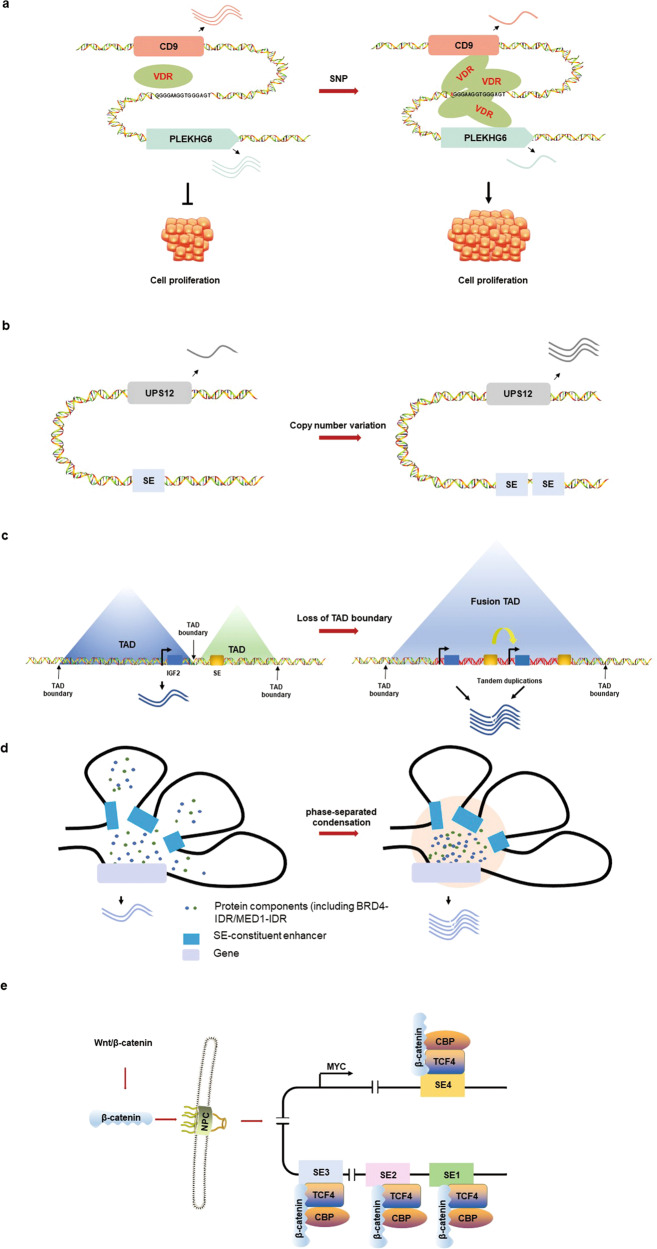
Formation and activation of SEs in colorectal cancer. **a** The SNP rs11064124G > A in a SE at chr12p13.31 promotes vitamin D receptor (VDR) binding, which downregulates the expression of the tumor suppressor genes *CD9* and *PLEKHG6* and thereby promotes the proliferation of CRC cells. **b** Focal amplification of SE on chr13q drives the high expression of UPS12 in CRC, which is a deubiquitinating enzyme implicated in prostate cancer. **c** Tandem duplications of the region between the *IGF2* locus and the SE on chromosome 11 disturb the TAD boundaries nearby, resulting in >250-fold overexpression of the *IGF2* gene. The yellow arrow represents the physical interaction between the promoter and SE of the *IGF2* gene, and the red DNA sequence represents the tandem duplicate region. **d** The IDRs of BRD4 and MED1 mediate the formation of phase-separated condensates at sites of SE-driven transcription, promoting the transcription of targeted genes. **e** The transcription of the MYC gene is regulated by multiple SEs, which are enriched with TCF4 binding sites. WNT signaling stabilizes and promotes the nuclear translocation of β-catenin, which forms a complex with TCF4 to activate *MYC* gene transcription in the nucleus. NPC, nuclear pore complex.

Genome-wide studies have shown that disease-related somatic variations occur primarily in noncoding sequences but are usually enriched in regulatory regions [52, 53]. Genetic alterations, including single-nucleotide polymorphisms (SNPs), insertions, deletions, genomic duplications, translocations, and inversions, can engender or inhibit SE formation through diverse mechanisms, resulting in disordered transcription of targeted genes functioning in cancers [54, 55]. The initiation and progression of tumors are often accompanied by specific gain or loss of related SEs. In CRC, the SNP rs11064124G > A in a cancer-specific SE at 12p13.31 promotes its binding affinity to vitamin D receptor (VDR), resulting in the greatly reduced expression of the tumor suppressor genes *CD9* and *PLEKHG6*, which leads to cancer cell proliferation (Fig. 1a) [56]. The SNP rs6854845 in an SE destroys the distant interaction between the SE and targeted gene clusters, affecting the transcription of these genes, which play important roles in colon cell growth and inflammatory responses [57]. SNPs were also found to regulate SE activities in neuroblastoma [58] and chronic lymphocytic leukemia [59], while short insertions introduced an MYB binding site in an SE in T cell acute lymphoblastic leukemia (T-ALL) [60]. In addition, copy number variations can result in the activation of oncogenic SEs. In CRC, focal amplification of SEs is one reason for aberrant oncogene expression; for example, UPS12 expression is upregulated by the chr13q amplicon (Fig. 1b) [61].

### Chromosomal remodeling and epigenetic regulation

Chromatin remodeling mainly refers to the dynamic rearrangement of chromatin architecture to allow access of condensed genomic DNA to the regulatory transcription machinery proteins, and thereby control gene expression. Topologically associating domains (TADs) are 3D structural units formed by chromatin loop architectures for transcriptional regulation, and their boundaries are usually determined by the CCCTC-binding factor (CTCF). TADs ensure proper physical interactions between promoters and distal enhancers/SEs, while the rearrangement of TADs has been proven to cause gene misexpression and disease [62, 63]. The destruction of the TAD boundary by somatic copy number alterations may change the TAD structure and lead to the formation of new TADs [64]. In CRC cells, tandem duplications of the *IGF2* locus were found to extend over the intervening TAD boundary, which enclosed an SE at the adjacent TAD and led to fusion TAD formation and *IGF2* overexpression (Fig. 1c) [65].

Epigenetic regulation through DNA and histone modifications plays an important role in the activation of SEs. Flavahan et al. showed that hypermethylation of CTCF binding sites compromised the binding of this methylation-sensitive insulator protein at TAD boundaries and thus permitted a constitutive enhancer to interact aberrantly with the receptor tyrosine kinase gene *PDGFRA* and activated its expression in IDH-mutant gliomas [66]. Lio et al. revealed that TET enzymes, which are dioxygenases that can promote DNA demethylation by oxidizing 5-methylcytosine to 5-hydroxymethylcytosine (5hmc), augmented activation-induced cytidine deaminase (AICDA) expression via 5hmc modifications in its SE in a mouse model [67].

In addition, SEs can move close to oncogene regulatory regions through chromosomal remodeling and cause corresponding oncogene activation, which is referred to as SE hijacking by oncogenes. For instance, a distant SE was found to be moved into proximity of the *MYB* gene via chromosomal translocations and activated its overexpression in adenoid cystic carcinoma [68]. In addition, the rearrangement of chromosome 3q results in distal *GATA2* enhancer translocation, which activates the expression of *EVI1* and causes functional haploinsufficiency of *GATA2* in leukemia, both of which are driven by chromosomal remodeling [69].

### Liquid–liquid phase-separated condensate and SE activation

Liquid–liquid phase separation (LLPS) of biological macromolecules, including nucleic acids and proteins, forms regional condensates or membraneless organelles in cells, which are sensitive to environmental cues and can exchange components in the cellular milieu, indicating that LLPS relates to dynamic, synergistic, and multivalent intermolecular interactions in cells [70]. A phase separation model has also been suggested to understand the underlying mechanisms of the formation, function, and characteristics of SEs due to its role in the regulation of gene transcription [71, 72]. Intrinsically disordered regions (IDRs) of proteins play a crucial role in the formation of membraneless organelles in LLPS [73]. Sabari et al. showed that the IDRs of BRD4 and MED1, two SE-associated transcription coactivators, mediated the formation of phase-separated droplets at the site of SE-related transcriptional apparatuses in nuclei, i.e., IDRs can play an important role in the compartmentalization and concentration of transcriptional components at specific SEs (Fig. 1d) [72]. Additionally, the activation domains in Mediator complexes and the master TFs OCT4 and GCN4 were found to be related to the initiation of phase-separated condensate formation [74]. The working model of transcriptional condensates that nucleate at SEs leading to chromatin reorganization for transcriptional regulation may also contribute to explaining SE biology in tumors.

### Abnormal transactivation and oncogenic signaling

Oncogenic SEs are usually rich in binding sites of key TFs that are regulated by tumor signaling pathways. Various oncogenic pathways and related TFs drive CRC development [75, 76]. CRC-related SEs were shown to be associated with MAPK, WNT, and TGF-β signaling [77]. For example, during the activation of the WNT signaling pathway, β-catenin accumulates in the cytoplasm and translocates into the nucleus, where it together with TCF4, which occupies the majority of CRC-driven SEs, strongly activates the transcription of the *c-MYC* gene, resulting in malignant progression (Fig. 1e) [78].

## The oncogenic roles and regulatory mechanisms of SEs in CRC

### Transcriptional regulation of targeted oncogenes

**Fig. 2:**
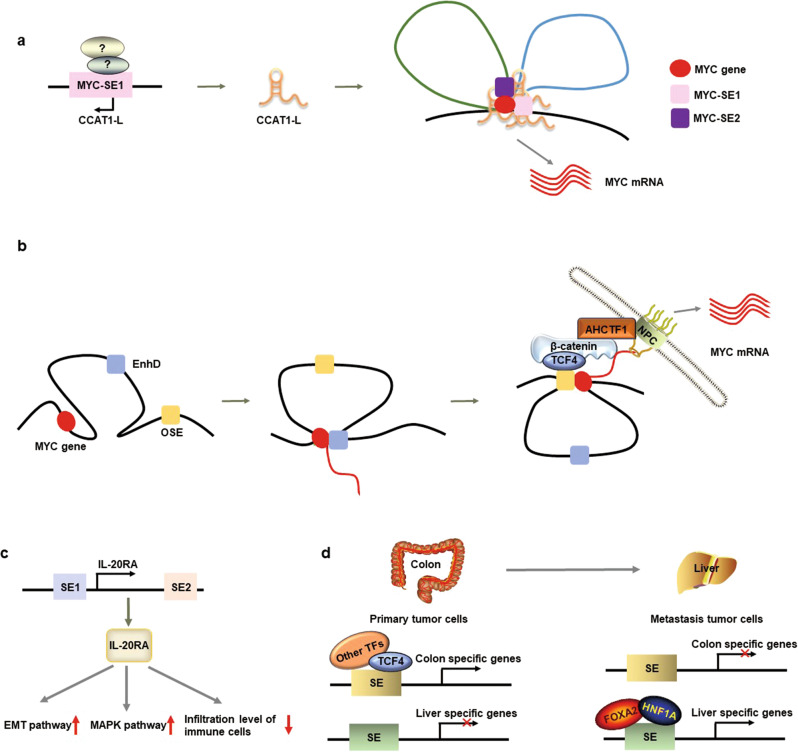
The oncogenic roles and regulatory mechanisms of SEs in colorectal cancer. **a** The long noncoding RNA CCAT-1L, derived from an MYC SE, mediates the interaction between SEs and the promoter of the *MYC* gene, regulating its transcription in collaboration with SEs. **b** AHCTF1 together with the β-catenin-TCF4 complex connects nucleoporins to the OSEs of the *MYC* gene, facilitating transcript export into the cytoplasm. **c** IL-20RA, transcriptionally regulated by several SEs, participates in oncogenic and immune pathways in CRC. **d** SEs participate in the liver metastasis of CRC via organ-specific transcription reprogramming.

SEs possess stronger abilities to regulate the transcription of their targeted genes than typical enhancers [12]. CRC-related SEs can promote the transcriptional expression of targeted oncogenes, causing the disorder of vital signaling pathways, such as those related to *c-MYC* [12, 78], *HOXB8* [79], and *IGF2* [65]. On the other hand, long noncoding RNAs (lncRNAs) derived from SEs can also regulate the expression of targeted oncogenes. For example, CCAT1-L, an lncRNA transcribed from a CRC-specific SE ~500 kb upstream of the *MYC* gene, mediates chromatin circularization between the promoter and SEs of the *MYC* gene, which increases gene transcription, thereby promoting the progression of CRC (Fig. 2a) [80]. AC005592.2, another SE-associated lncRNA, regulates the proliferation, migration, and invasion of CRC by upregulating the transcription of OLFM4 [81].

### Gene gating regulation of oncogene expression

The efficiency of oncogene expression can be further facilitated by SE-mediated transcriptional regulation combined with other mechanisms. MYC is a typical oncogene controlled by SE-mediated transcription-regulating mechanisms, and WNT signaling activates MYC expression in cancer cells (Fig. 1e). Scholz et al. demonstrated that WNT signaling and AHCTF1 promoted oncogenic MYC expression posttranscriptionally through a mechanism termed SE-mediated gene gating [82]. This involves oncogenic SE-mediated tethering of active MYC alleles to nuclear pores to increase transcript export into the cytoplasm and help transcripts escape the nuclear degradation system in colon cancer cells; in this process, AHCTF1 connects nucleoporins to the OSE via the β-catenin-TCF4 complex (Fig. 2b). In comparison, EnhD (a representative nononcogenic SE) does not bind to nucleoporins and only interacts with the *MYC* promoter to regulate transcription in the nucleus.

### Regulation of immune evasion

Immune evasion facilitates tumorigenesis and tumor metastasis. IL-20RA, transcriptionally controlled by its SEs, contributes to invasion and metastasis in carcinoma progression in CRC by regulating some oncogenic pathways and immune response pathways, which results in a decrease in the infiltration of N1 neutrophils and M1 macrophages as well as the recruitment of T cells (Fig. 2c) [83].

### Reprograming of organ-specific gene expression

Metastasis and recurrence are responsible for the vast majority of tumor mortality. Transcriptional reprogramming helps metastatic cancer cells better adapt to their new environments. Gene expression profiling of primary CRC and liver-metastatic samples revealed that a liver-specific gene program appeared while the CRC-specific program disappeared in liver-metastatic CRC cells, even before their colonization of the liver. Specifically, transcription reprogramming was driven by epigenetic mechanisms associated with enhancers and SEs, which were enriched with the binding sites for the liver-specific TFs FOXA2 and HNF1A, thereby promoting CRC liver metastasis (Fig. 2d) [84]. The reprogramming of organ-specific gene expression before metastasis has also been identified in some other types of primary tumors, including lung-metastatic CRC, bone-metastatic prostate cancer, liver-metastatic pancreatic cancer, and brain-metastatic breast cancer. Therefore, enhancer and SE profiles of primary tumors may help to predict cancer metastasis and patient survival.

## Therapeutic strategies and compounds targeting oncogenic SEs in CRC and other malignancies

### Targeting SE-related transcription machinery

Targeting oncogenic transcription programs is an attractive anticancer strategy; however, a significant challenge is the selection of inhibitors that can specifically target oncogenic components in cancer cells with minimal toxicity in normal cells [32]. Oncogenic SEs and related transcriptional regulators can specifically control tumor cell fate and thereby have been vigorously explored as novel potential targets for cancer therapy in recent years. The initiation, pausing and elongation of transcription tend to proceed via sequential activation of regulatory and enzymatic cofactors. During the process, active oncogenic SEs, marked by H3K27ac, which is recognized by BRD4, interact with the complex of Mediator coactivators; this is followed by the stepwise recruitment of TFIIH, the CDK7-containing initiation complex, and P-TEFb, the CDK9-containing elongation complex [32]. These components that function in the SE-related core transcription machinery have been investigated as therapeutic targets for the inhibition of oncogenic transcription [19, 28, 30]. Among them, some transcriptional and epigenetic inhibitors, such as BET inhibitors and CDK7 and CDK9 inhibitors, have shown encouraging antitumor potential in preclinical experiments or clinical trials for various malignancies [85–96] (Table 2).

**Table 2:** SEs-targeting therapeutic inhibitors in clinical trials.

Target	Inhibitor	Mechanism	Tumor type	Clinical trial
BET proteins	BMS-986158	Decreasing BRD4 occupation and MED1 binding on SEs by blocking BD1 or BD2 of BRD4	Advanced Solid Tumors	NCT02419417 (phase I/IIa)
			Hematologic Malignancies	
	OTX015 (Birabresib)		AML GMA	NCT02303782 (phase II)
				NCT02296476 (phase II)
	GSK525762		Neoplasms	NCT01943851 (phase II) / [87]
	CPI-0610		PNTs MF Neoplasms	NCT02986919 (phase II)
				NCT04603495(phase III)
				NCT02158858 (phase II)
	AZD5153	Decreasing BRD4 occupation and MED1 binding on SEs by blocking BD1 and BD2 of BRD4	Malignant Solid Tumors Lymphoma	NCT03205176 (phase I)
CDK7	CT7001	Blocking TFIIH function by non-covalent binding to the ATP-binding site of CDK7	Advanced Solid Malignancies	NCT03363893 (phase I/II)/ [88]
	SY-5609		Advanced Solid Tumor, BC, SCLC	NCT04247126 (phase I)/ [88]
	SY-1365	Blocking TFIIH function by covalent binding to the ATP-binding site of CDK7	Ovarian cancer, breast cancer, advanced solid tumors	NCT03134638 (Phase I)
	LY3405105	/	advanced or metastatic solid cancers	NCT03770494 (phase Ia/Ib)
CDK9	Fadraciclib	Blocking P-TEFb function by inhibiting the ATP-binding site of CDK9	Solid Tumor, Lymphoma MDS	NCT04983810 (phase II)
				NCT03593915 (phase II)
	Dinaciclib		CLL	NCT01580228 (phase III)/ [89]
	Alvocidib		MDS, Secondary MDS AML Malignant Solid Tumor	NCT03593915 (Phase I)
				NCT03441555 (Phase I)
				NCT03604783 (Phase I)
	AZD4573		Advanced hematological cancers and relapsed/refractory hematological cancers	NCT03263637 (Phase I)
	BAY-1143572 (Atuveciclib)		Acute leukemias and advanced malignancies	NCT02345382 (Phase I)
				NCT01938638 (Phase I)
	BAY-1251152		Advanced hematological cancers and advanced malignancies	NCT02745743 (Phase I)
				NCT02635672 (Phase I)

Abbreviations: Acute Myeloid Leukemia (AML), Glioblastoma Multiforme (GMA), Peripheral Nerve Tumors (PNTs), Myelofibrosis (MF), Myelodysplastic Syndromes (MDS), Breast Cancer (BC), Small-cell Lung Cancer (SCLC), Chronic Lymphocytic Leukemia (CLL).

### BET inhibitors

Bromodomain (BRD) proteins, including BRD4, are epigenetic readers of histone acetylation involved in chromatin remodeling and transcriptional regulation. Inhibition of bromodomain and extraterminal (BET) family proteins preferentially causes the loss of BRD4 occupancy at SEs [14], leading to antitumor effects in vitro and in vivo [93]. Small-molecule inhibitors of BET proteins can be divided into monovalent inhibitors (e.g., JQ1, OTX015, GSK525762, and CPI-0610) and bivalent inhibitors (e.g., AZD5153 and MT1). In CRC, JQ1 is the most studied BRD4 inhibitor preclinically, and OTX015 was optimized based on JQ1. Several studies have shown that SEs can be sensitive or resistant to BET inhibitors, while rational combinations with oncogenic pathway inhibitors will enhance the therapeutic potential and reduce the side effects of BET inhibitors in CRC [97]. Tögel et al. showed that JQ1 selectively bounds to the acetyl-lysine recognition domain of BRD4, and CRC cells with microsatellite instability were more sensitive to it [98]. JQ1 treatment combined with the inhibition of the WNT/β-catenin/TCF signaling pathway by β-catenin siRNAs or with the inhibition of the MEK/ERK pathway by the MEK inhibitor trametinib more significantly downregulated the expression of c-MYC and induced a more potent antiproliferative effect than single treatment in CRC cells [98]. Another study by Yoshiaki et al. revealed that a BET inhibitor combined with a MEK inhibitor effectively overcame the intrinsic resistance to JQ1 and repressed the growth of colon cancer cells by further decreasing the expression of c-MYC [99]. Interestingly, McCleland et al. showed that JQ1 caused growth arrest and differentiation in a subset of colon cancers characterized by the CpG island methylator phenotype (CIMP), and c-MYC transcription was very dependent on BET activity in these colon cancers. The expression of CCAT1, an lncRNA transcribed from a distinct c-MYC SE in CIMP + colon cancers, predicted JQ1 sensitivity and BET-mediated c-MYC transcription, suggesting it as a clinically tractable biomarker for identifying patients who will likely benefit from BET inhibitors [100].

Nevertheless, another study showed that colon cancer-specific SEs were associated with the MAPK signaling pathway, and the sensitivity to JQ1 was not related to c-MYC expression among 14 colon cancer cell lines. The combination of JQ1 with vemurafenib, an inhibitor of BRAF V600E, repressed cell growth by inducing cell cycle arrest and apoptosis in BRAF^V600E^-mutant cells. Mechanistically, JQ1 suppressed the feedback activation of EGFR by vemurafenib, which participates in the MAPK signaling pathway [77].

One of the resistance mechanisms to BET inhibitors is paracrine IL6/IL8-JAK2 signaling in CRC, which induces the phosphorylation of BRD4 at tyrosine 97/98, increasing the binding capacity of BRD4 to chromatin but reducing that to BET inhibitors. Interruption of IL6/IL8-JAK2 signaling suppressed the phosphorylation of BRD4 and increased sensitivity to BET inhibitors in vitro and in vivo [101]. The stromal mechanism underlying the activation of BRD4 and resistance to BET inhibitors suggests that a rational combinatorial strategy will be more effective for the treatment of CRC.

### Inhibition of transcription-regulating CDKs

In addition to BET inhibitors, inhibition of CDK7 and CDK9 can be considered another potential approach for targeting oncogenic SE-involved transcription because of the function of these proteins in regulating RNA Pol II initiation and elongation, respectively [102]. THZ1, a specific covalent inhibitor of CDK7, inhibited the phosphorylation of the carboxyl-terminal domain (CTD) of RNA Pol II, resulting in the inhibition of transcriptional initiation [103]. THZ1 has shown antitumor activity by targeting SE-associated transcription in preclinical studies in various cancers, such as SE-driven MYCN neuroblastoma [92], SE-driven RUNX in T-ALL [94], triple-negative breast cancer [91], and small-cell lung cancer [104]. SY-1365 (a THZ1 derivative), a selective inhibitor of CDK7 under clinical trials in breast and ovarian cancers, preferentially decreased the expression of SE-related oncogenic genes with very little influence on housekeeping genes in acute myeloid leukemia (AML) cells [105]. SEs can be transcribed into enhancer RNAs (eRNAs) [106], which are correlated with the expression of nearby genes, and the treatment of cells with the CDK9 inhibitor alvocidib led to a decrease in eRNA transcription elongation. Moreover, A51, a small-molecule inhibitor cotargeting CKIa and CDK7/9, abrogated several SE structures and repressed the transcriptional elongation of SE-driven oncogenes, synergistically stabilizing P53 [107]. In chordoma cells, inhibition of CDK7 by THZ1 or CDK9 by NVP-2 resulted in the downregulation of SE-related brachyury/TBXT (a developmental and oncogenic TF) in a preferential and concentration-dependent manner [108].

### Targeting epigenetic modifiers

Posttranslational modification of histones is important to chromatin architecture and gene transcriptional regulation. H3K27me3 levels, distorted in the vast majority of human cancers, are regulated by polycomb complex 2 (PRC2) and lysine demethylase 6 (KDM6) family proteins. In CRC, targeted inhibition of KDM6 by GSK-J4, an inhibitor of KDM6 histone demethylases, effectively eradicated tumor-initiating cells and downregulated the stemness-associated signature genes ID1 and TERT. Mechanistically, KDM6 inhibition induced global enhancer reprogramming with a preferential impact on SE-associated genes; for example, it decreased the level of H3K27ac and increased the levels of H3K4me1, p300, BRD4, and KDM6A at the ID1 locus [109].

## Concluding remarks

Although the similarities and differences in definition, composition, and functional significance among SEs, enhancers that may comprise SEs, and previously defined transcriptional regulatory genomic regions need more investigation and validation for further clarification, it has been demonstrated that oncogenic SEs play important roles in carcinogenesis and malignant progression in a context-dependent manner. In CRC, cancer cells can form oncogenic SEs through genetic and epigenetic alterations and 3D chromosomal remodeling, and CRC-related SEs can activate the expression of oncogenes via transcriptional and posttranscriptional mechanisms, facilitate immune escape and propel cancer proliferation and metastasis. SE-related mechanisms possess tissue, cell, and/or cancer-type specificity. However, the cancer-specific and cancer-type-related composition, dynamic assembly and functional activation of oncogenic SEs, and their underlying molecular mechanisms driving cancer development, still need more studies in the future.

To date, many small-molecule inhibitors targeting SE-related transcriptional components have been evaluated in preclinical experimental models and clinical trials and have shown promising activities against multiple types of advanced cancers. Although recently clinically evaluated targets, such as BRD4, the Mediator complex, and the CDK7-TFIIH and CDK9-pTEFb complexes, are common binding proteins on transcriptional regulatory elements, they are highly enriched in oncogenic SEs; therefore, their inhibition preferentially impacts genes with oncogenic SEs and shows relative selectivity to cancer cells. In the future, further dissecting and characterizing gene-specific SE complex components and related underlying mechanisms will help to discover novel cancer-specific therapeutic targets and more selective and potent drugs for cancer treatment.

## Data Availability

All data generated or analyzed during this study are included in this published article.
